# Retraction: MTA1 promotes hepatocellular carcinoma progression by downregulation of DNA-PK-mediated H1.2^T146^ phosphorylation

**DOI:** 10.3389/fonc.2026.1839585

**Published:** 2026-05-13

**Authors:** Frontiers Editorial Office

The journal retracts the May 6^th^ 2020 article cited above.

Following publication, concerns were raised regarding the integrity of the images in the published figures. There were concerns about the multiple images in Figures 2, 5 and 7, which also appeared in another publication from a different publisher, but with different labelling. The authors have remained unresponsive to multiple requests for the raw data for this study. As a result, the data and conclusions of the article have been deemed unreliable, and the article has been retracted.

The investigation followed Frontiers’ policies, and the retraction was approved by the Chief Executive Editor. The authors were informed about the retraction.

